# Evaluating the Quality of Cancer-Related WeChat Public Accounts: Cross-Sectional Study

**DOI:** 10.2196/52156

**Published:** 2024-05-30

**Authors:** Peng Pan, Changhua Yu, Tao Li, Tingting Dai, Hanhan Tian, Yaozu Xiong, Jie Lv, Xiaochu Hu, Weidong Ma, Wenda Yin

**Affiliations:** 1 Department of Radiation Oncology The Affiliated Huaian No.1 People's Hospital of Nanjing Medical University Huai'an China

**Keywords:** cancer, big data, social media, health literacy, WeChat, China, public health

## Abstract

**Background:**

WeChat (Tencent) is one of the most important information sources for Chinese people. Relevantly, various health-related data are constantly transmitted among WeChat users. WeChat public accounts (WPAs) for health are rapidly emerging. Health-related WeChat public accounts have a significant impact on public health. Because of the rise in web-based health-seeking behavior, the general public has grown accustomed to obtaining cancer information from WPAs. Although WPAs make it easy for people to obtain health information, the quality of the information is questionable.

**Objective:**

This study aims to assess the quality and suitability of cancer-related WeChat public accounts (CWPAs).

**Methods:**

The survey was conducted from February 1 to 28, 2023. Based on the WPA monthly list provided by Qingbo Big Data, 28 CWPAs in the WeChat communication index were selected as the survey sample. Quality assessment of the included CWPAs was performed using the HONcode instrument. Furthermore, suitability was measured by using the Suitability Assessment of Materials. A total of 2 researchers conducted the evaluations independently.

**Results:**

Of the 28 CWPAs, 12 (43%) were academic and 16 (57%) were commercial. No statistical difference was found regarding the HONcode scores between the 2 groups (*P*=.96). The quality of the academic and commercial CWPAs evaluated using the HONcode instrument demonstrated mean scores of 5.58 (SD 2.02) and 5.63 (SD 2.16), respectively, corresponding to a moderate class. All CWPAs’ compliance with the HONcode principles was unsatisfactory. A statistically significant difference between the 2 groups was observed in the Suitability Assessment of Materials scores (*P*=.04). The commercial WPAs reached an overall 55.1% (SD 5.5%) score versus the 50.2% (SD 6.4%) score reached by academic WPAs. The suitability of academic and commercial CWPAs was considered adequate.

**Conclusions:**

This study revealed that CWPAs are not sufficiently credible. WPA owners must endeavor to create reliable health websites using approved tools such as the HONcode criteria. However, it is necessary to educate the public about the evaluation tools of health websites to assess their credibility before using the provided content. In addition, improving readability will allow the public to read and understand the content.

## Introduction

According to the International Agency for Research on Cancer (IARC), cancer is the first or second leading cause of premature mortality in over 90 countries. China accounts for 23.7% of global new cases and 30% of deaths. In China, the age-standardized incidence and mortality rates of all cancers were 201.7 per 100,000 and 130.1 per 100,000 in 2018, respectively [1]. In recent decades, the cancer burden in China has increased, posing a serious threat to public health.

Moreover, with the rapid development of the internet, social media has remarkably changed people’s lifestyles [2]. Similar to Facebook, WeChat, released in 2011 by Tencent Inc, has become the most widely used social networking platform in China, reporting 1299 million users in 2022 [3]. WeChat public accounts (WPA) are application accounts supplied by administrators that can be used for communication and interaction with specific groups via text, pictures, videos, and so forth. Members can follow the WPAs of interest to receive relevant information or messages. In early 2020, more than 1 million papers were posted daily on WeChat [4]. WeChat is one of the most important sources of information for the Chinese public. Pertinently, various health-related information is continuously transmitted among WeChat users. Health-related WeChat public accounts (HWPAs) are being rapidly developed [5]. HWPAs have an important impact on public health status. Although it is convenient for people to obtain health information from WPAs, the quality of the health information is questionable [6]. Therefore, it is important to evaluate the quality of the HWPAs. Previous studies have explored the use of WPAs in health education [7-9]. However, few have focused on the quality of HWPAs. Wang et al [10] examined 93 HWPAs to evaluate their quality and found that they were substandard according to the Net Foundation Code of Conduct (HONcode) conformity. Furthermore, there is still a lack of general understanding regarding the quality of cancer-related WeChat public accounts (CWPAs). Owing to increasing web-based health-seeking behaviors, the public has become accustomed to obtaining cancer information through WPAs. Therefore, this study was conducted to evaluate the quality of CWPAs. Our study aims to assess the quality and suitability of CWPAs.

## Methods

### Data Collection

The data used in this study were derived from the Qingbo Big Data platform, the largest third-party evaluation platform for new media in China. Qingbo Big Data Technology Co, Ltd (Beijing, China) was established in October 2014. The company provides big data technology services to the Chinese government, top Chinese news media, and large multinational enterprises [11]. The WeChat communication index (WCI), proposed by Qingbo Big Data, is the most widely used standard for evaluating the influence of WPAs [10]. The WCI comprises 4 primary indicators (the overall paper spread rate, average spread rate of each paper, title spread rate, and peak spread rate), 8 secondary indicators, and a set of calculation formulas for standardized scores [12]. A higher WCI value indicates a larger WPA influence. We searched for new media in the cancer category of the WPA monthly list (February 1 to 28, 2023) provided by Qingbo Big Data. The CWPAs in the WCI were selected as the survey sample. The exclusion criteria for CWPAs were as follows: (1) having been completed for commercial purposes, and (2) no papers released during the survey period. As a result, 18 CWPAs were excluded according to the criteria. Finally, 28 CWPAs were included in this study (Multimedia Appendix 1). We analyzed 1503 papers released by each CWPA on the survey dates.

### Evaluation Tools

Quality assessment of the included CWPAs was performed using the HONcode instrument. Health on the Internet is an independent organization that provides health information guidelines for websites based on 8 principles: authoritativeness, complementarity, privacy, attribution, justifiability, transparency, financial disclosure, and advertising policies [13] (Multimedia Appendix 2). The introduction of the HONcode in 1996 was a milestone for web-based health information, as evidenced by the numerous references to the HONcode in the Health Informatics literature. The HONcode has often been used as a major indicator of content accuracy in scientific studies [14]. As CWPAs are used to disseminate cancer-related health knowledge to the public, these WPAs should also comply with the HONcode principles. Thus, we believe analyzing the credibility and reliability of the information on CWPAs using the HONcode instrument is appropriate. We adopted a similar HONcode scoring system to that previously published [15]. For each CWPA, the respect or no respect to each HONcode principle was scored as 0 (nonconformity) or 1 (conformity). As a result, the quality of the CWPAs was classified as low (HONcode 0-2), moderate (HONcode 3-5), or high (HONcode 6-8).

The Suitability Assessment of Materials (SAM) created by Doak et al [16] was designed to assess educational material. Applying the SAM can pinpoint specific deficiencies in suitability, and if the material is still in the developmental stage, these deficiencies can be corrected. The SAM comprises 22 criteria in 6 categories: content, literacy demand, graphics, layout and typography, learning stimulation and motivation, and cultural appropriateness (Multimedia Appendix 3). Within these categories, according to how well they meet the criteria for each item, individual items are rated as follows: not applicable, 0 (not suitable), 1 (adequate), or 2 (superior). The sum of the ratings obtained was divided by the total possible score and transformed into percentages. A total of 3 levels are used to categorize the percentage score: 70%-100%, “superior”; 40%-69%, “adequate”; and 0%-39%, “not suitable” [16]. The SAM has been tested and validated in individuals of various cultural backgrounds [17]. In a study by Chang et al [18], the SAM was proven valid and reliable for evaluating the suitability of health-education materials in Chinese. Therefore, in this study, we used the SAM to evaluate health information released by CWPAs. These CWPAs were classified into academic WPAs and commercial WPAs according to a study by Valizadeh-Haghi et al [19]. The findings of this study revealed that there was a significant association between the website category and the credibility of health websites.

A total of 2 researchers conducted the evaluations. These 2 raters independently evaluated CWPAs’ compliance with the principles of the HONcode and the suitability of the papers released by the CWPAs using the SAM scale. Any controversial assessment results were resolved through real-time negotiations. Cohen κ test assessed interrater reliability, with a score of 0.83 indicating almost perfect agreement [20].

### Statistical Analysis

Statistical analysis was performed using SPSS (version 26.0; IMB Corp). Numerical variables are reported as mean (SD) or median (IQR) values. We tested the normality of the distribution of the numerical variables using the Shapiro-Wilk test before proceeding with a parametric or nonparametric test. Parametric variables were compared using the Student *t* test and nonparametric continuous variables were evaluated with the Mann-Whitney *U* test. Categorical variables were presented as numbers (n) and percentages (%). Fisher exact test was used to compare categorical variables. Statistical significance was set at *P*<.05.

### Ethical Considerations

According to Article 32 of the ethical review guideline of life science and medical research, which was issued by the National Health Commission of the People’s Republic of China on February 18, 2023, because only publicly available data were involved in our study, the ethical review could be exempted [21]. All data were anonymized.

## Results

### Characteristics of the CWPAs

The general characteristics of the CWPAs are listed in Table 1. Of the 28 CWPAs, 12 (43%) were academic and 16 (57%) were commercial. A statistically significant difference between the 2 groups was observed in the SAM scores (*P*=.04). The commercial WPAs reached an overall 55.1% (SD 5.5%) score versus the 50.2% (SD 6.4%) score reached by academic WPAs. The suitability of academic and commercial CWPAs was considered adequate. Statistical differences were also found regarding views (*P*=.04), likes (*P*=.03), and WCI (*P*=.03). Notably, the numbers of views, likes, and WCI were higher for commercial WPAs than for academic WPAs. Although no statistical difference was found regarding the HONcode scores between the 2 groups (*P*=.96), commercial WPAs seemed more compliant than academic WPAs. The quality of the academic and commercial CWPAs evaluated using the HONcode instrument demonstrated mean scores of 5.58 (SD 2.02) and 5.63 (SD 2.16), respectively, corresponding to a moderate class.

**Table 1:** Analysis of cancer-related WeChat public account characteristics by ownership.

Characteristics	Academic CWPAs^a^	Commercial CWPAs	*P* value
Number of papers	322	1181	N/A^b^
Number of views, median (IQR)	59,109 (1534.2-60,643.2)	420,237.75 (19,762-439,999.75)	.04
Number of likes, median (IQR)	226.25 (5.25-231.5)	1640.25 (82.75-1723)	.03
WCI^c^, mean (SD)	453.57 (255.46)	700.09 (300.20)	.03
HONcode scores, mean (SD)	5.58 (2.02)	5.63 (2.16)	.96
SAM^d^ scores (%), mean (SD)	50.2 (6.4)	55.1 (5.5)	.04

^a^CWPA: cancer-related WeChat public account.

^b^N/A: not applicable.

^c^WCI: WeChat communication index.

^d^SAM: Suitability Assessment of Materials.

### Health on the Net Foundation Code of Conduct Conformity

The HONcode compliances of the 28 CWPAs according to ownership are listed in Table 2. Except for the advertising principle (*P*=.02), there were no statistical differences regarding the other 7 principles between the academic and commercial groups. CWPAs’ compliance with the HONcode principles was not ideal. Most academic and commercial WPAs failed to meet the principles of transparency and financial disclosure (58% vs 62% and 58% vs 69%, respectively). One-third of academic (4/12, 33%) and almost half of the commercial WPAs (7/16, 44%) did not respect this attribution principle. All academic WPAs and 94% (n=15) of the commercial WPAs received a full score on the justifiability principle. Compliance was also uneven for authoritative and complementarity principles. A greater proportion of academic WPAs achieved full scores in the authoritative and complementarity principles (10/12, 83% and 11/12, 92%, respectively), compared with commercial WPAs (11/16, 69%). Finally, only 25% (n=3) of academic WPAs received a full score in the advertising policy principle, compared with commercial WPAs (12/16, 75%); that is, three-quarters of the academic WPAs did not clearly distinguish advertising from editorial content.

**Table 2:** Evaluating HONcode scores according to ownership.

HONcode principles	Proportion of academic CWPAs^a^ with full score (n=12), n (%)	Proportion of commercial CWPAs with full score (n=16), n (%)	*P* value
Authoritative	10 (83)	11 (69)	.66
Complementarity	11 (92)	11 (69)	.19
Privacy	12 (100)	16 (100)	N/A^b^
Attribution	8 (67)	9 (56)	.71
Justifiability	12 (100)	15 (94)	.38
Transparency	5 (42)	6 (38)	.82
Financial disclosure	5 (42)	5 (31)	.69
Advertising policy	3 (25)	12 (75)	.02

^a^CWPA: cancer-related WeChat public account.

^b^N/A: not applicable.

### Suitability of Papers From WPAs

Table 3 presents the analysis of the CWPAs’ readability using the SAM. Among the 6 categories, a statistically significant difference was found between academic and commercial WPAs in literacy demand (*P*=.02). In most cases, the mean scores of academic WPAs were lower than those of commercial WPAs, except for the cultural appropriateness items. However, no statistical differences were found between the 2 groups regarding content (*P*=.53), graphics (*P*=.07), layout and typography (*P*=.84), learning stimulation and motivation (*P*=.95), or cultural appropriateness (*P*=.78). None of the CWPAs achieved a superior score on the SAM items. The percentages of criteria met in each of the 6 SAM categories ranged from the lowest for learning stimulation and motivation to the highest for content.

**Table 3:** Evaluating Suitability Assessment of Materials scores of papers on the cancer-related WeChat public account according to ownership.

SAM^a^ items	Academic CWPAs^b^, mean (SD)	Commercial CWPAs, mean (SD)	*P* value
Content (purpose is evident, content regarding behavior, scope is limited, and summary or review included)	5.08 (0.51)	5.25 (0.86)	.53
Literacy demand (reading grade level, writing style, active voice, vocabulary uses common words, context is given first, and learning aids via “road signs”)	4.58 (0.79)	5.56 (1.15)	.02
Graphics (cover graphic shows purpose; type of graphics; relevance of illustrations; list, tables, etc explained; and captions used for graphics)	4.67 (1.15)	5.63 (1.41)	.07
Layout and typography (layout factors, typography, and subheads used)	3.83 (0.58)	3.88 (0.50)	.84
Learning stimulation and motivation (interaction used, behaviors are modeled and specific, and motivation [self-efficacy])	1.67 (0.98)	1.69 (0.79)	.95
Cultural appropriateness (match in logic, language, and experience; cultural image; and examples)	2.25 (0.62)	2.19 (0.54)	.78

^a^SAM: Suitability Assessment of Materials.

^b^CWPA: cancer-related WeChat public account.

In the content category, most WPAs stated their purpose in the titles and contained related information within the necessary scope. However, some (8/28, 28.6%) of the WPAs contained behavior-related context in presenting content; that is, the content mainly included facts about cancers and not guides for readers’ behavior or decision-making. Additionally, 14% (n=4) of WPAs did not include a summary or review. Regarding literacy demand, 1 WPA was classified as not suitable, 26 as adequate, and only 1 was superior. The cover graphics for most papers released by the WPAs were rated as superior. However, 68% (n=19) of the WPAs used illustrations inappropriately. Moreover, one-quarter (7/28, 25%) of the papers did not include captions that detailed the information in the tables and graphs. The layouts of most papers received high scores; for example, most were adequate and superior regarding typography and font size. Lower ratings were caused by the tendency to include too much information under the subheadings. The learning stimulation and motivation categories had the lowest ratings. None of the studies provided web-based learning stimulation. The content for behavioral modeling and self-efficacy of 93% (n=26) of the WPAs was adequate or not suitable. Most WPAs (26/28, 93%) were rated as adequate or superior for using positive images and examples for the cultural appropriateness category.

## Discussion

### Principal Findings

WeChat is the most popular platform for acquiring health information. Health information acquisition via WeChat is more convenient, timely, and cost-effective; moreover, it protects privacy and avoids embarrassment. Furthermore, the technical development of big data and the Internet of Things allows individuals to access, track, and customize health information. To a certain extent, WeChat contributes to greater freedom regarding individual health decisions.

The public encounters problems through the internet via an overload of information. In our study, searching for “cancer” in the Qingbo search engine generated 46 WPAs. Worryingly, the information presented in these WPAs is not sufficiently credible; that is, valid and valuable information is obscured by irrelevant and misleading information. To our knowledge, this was the first study to evaluate the quality and readability of WPAs concerning cancer. Our study mirrored other studies’ findings on various topics [22-24].

The HONcode instrument for health-related web resources has been available for 20 years. A failure to comply with the HONcode criteria indicates that users may encounter websites that are not sufficiently reliable. These websites may contain inaccurate, misleading, and inadequate information, which can influence preventive actions and decision-making regarding cancer treatment choices.

This study’s findings revealed that all CWPAs’ compliance with the HONcode principles was unsatisfactory. Although one could intuit that the information found in academic CWPAs would yield the highest quality information, our study found this was not always true. Specifically, there was no statistical difference between academic WPAs and commercial CWPAs regarding HONcode sum scores and most HONcode categories. Thus, academic institutions must take substantial steps to improve the credibility of their WPAs to comply with the HON principles.

Compliance with the authority criterion reflects the credibility of the information source because this principle proves that the information provided by experts is reliable [25]. In this study, one-quarter (7/28, 25%) of the surveyed CWPAs did not specify the names or expertise of the authors. In a similar study evaluating Persian language health websites on Ebola, the authorities obtained the lowest score [22]. While the public needs sufficient information about the author’s identity to assess the trustworthiness of information, CWPAs must pay more attention to this criterion to increase trustworthiness for their readers. The complementarity aspect of web-based medical information should be clearly stated on health websites as such information is intended to provide support and training for readers and should not be a substitute for direct medical advice [19]. Nevertheless, 21.4% (n=6) of the surveyed CWPAs did not consider this criterion, which may have led to misuse of information. Moreover, CWPAs should describe their privacy policies and define how they handle users’ private information such as email addresses and content. This policy is among the 7 core issues in website usability design and is particularly important for creating effective websites [26]. Satisfactorily, all CWPAs assessed in this study identified their privacy policies. According to the attribution principle, the publication date and most recent content updates should be posted on the website. Adherence to this principle can ensure the credibility of health websites. This study revealed that the attribution principle was considered in more than half (17/28, 61%) of the CWPAs. Nevertheless, 39% (n=11) of the CWPAs did not pay sufficient attention to this principle. The justifiability criterion indicates that any information on a website must support claims regarding the benefits or performance of a particular treatment, medication, or medical device. Overall, in this study, the adherence to the justifiable principle was good. The transparency principle states that when additional information is required, people must be able to connect with content editors and communicate with webmasters. Unfortunately, based on the present findings, this principle was only considered in 39% (n=11) of the CWPAs. Financial disclosure and advertising principles imply that there should be a clear distinction between commercial and scientifically edited content presented on CWPAs. If advertising is a source of funding for a WPA, the financial disclosure policy for presenting such content should be clearly stated. Moreover, failure to comply with advertising policies indicates that individuals may be unable to distinguish advertisement information from the main content. Access to such WPAs may guide readers toward unreliable information that may threaten their health. However, only 36% (n=10) of the surveyed CWPAs considered financial disclosure principles. More importantly, 75% (n=9) of the academic WPAs failed to comply with the advertising policy. In contrast, only 25% (n=4) of commercial WPAs failed to comply with this principle.

The content provided to the public must not only meet the reliability standard but also be at the required reading level that allows people of all educational levels to understand and process information related to their disease and treatment options [27]. Notably, lower overall health literacy is associated with increased complications, hospitalizations, poor understanding of the disease, and increased health care costs [28]. This study determined the CWPAs’ information suitability to be “adequate.” Cultural and linguistic differences inevitably lead to differences in people’s health-related behaviors and understanding of web-based health information. Thus, website owners must consider additional acculturation factors when publishing health information. However, this creates higher requirements for user cultural literacy [11].

Regarding scoring dimensions, most papers published by the CWPAs had appropriate cover pictures and attractive titles that clearly described the paper’s purpose, a good layout and typography, and were culturally suitable. However, the nonstandard use of charts and the lack of charts used as illustrations were common problems. More than half of the papers included pictures with weak relevance to the content of the papers or even harmful overstatements and stereotypical cultural characteristics. In addition, regarding vocabulary, readers had difficulty reading papers generated by professional WPAs because they usually use more scientific terms.

The cultural appropriateness of health-education materials is enhanced when readers view illustrations and graphics that are easily recognizable and depict people similar to themselves and those around them. Many factors affect health care including cultural beliefs and practices [17]. Thus, it is important to consider these factors when designing health education materials.

We are particularly interested in examining the facilitation of self-efficacy. Applying the concept of self-efficacy is an effective means of promoting positive health behaviors and informed decision-making. Self-efficacy theory explains and predicts how people influence their motivation and behavior; to enhance self-efficacy, materials must model the desired behavior using someone similar to the intended audience [17]. Very few of the reviewed materials used appropriate methods to enhance readers’ self-efficacy.

Based on our findings, although the suitability of health information released by CWPAs was at a moderate level, the overall quality of accessible information on CWPAs was inadequate. Failure to comply with all HONcode criteria in these CWPAs shows that while searching for WPAs, users will encounter impressive websites, and consequently, low-quality information that can affect their health care practices for cancer. Reliable and readable information is essential for overcoming the potential negative aspects of web-based health information. Providing information in shorter sentences with simple words and using figures or videos may help improve the public’s understanding of cancer and cater to people with varying levels of health literacy. This highlights the importance of understanding the quality of CWPAs by providers and guiding the public toward reliable sources. Finally, it is recommended that the papers of CWPAs be subjected to some form of peer review, similar to those used for journal paper submissions, before the final upload. This would create a core set of high-quality, publicly available information.

### Limitations

This study has some limitations. The study was conducted between February 1 and 28, 2023; therefore, it does not completely and comprehensively represent other studies conducted at different times. However, owing to the dynamic characteristics of the web, search results vary at different times and places. New websites are constantly being created, while some websites are being disbanded. Second, there are many evaluation indices for WPAs; however, horizontal comparisons of these indices are lacking. We chose the WCI proposed by Qingbo Big Data as the ranking basis for the influence of WPAs, which may have resulted in selection bias. Finally, this study was conducted only on Chinese websites. Therefore, the results of this study may differ from those conducted in other languages.

### Conclusions

This study revealed that CWPAs are not sufficiently credible. WPA owners must endeavor to create reliable health websites using approved tools such as the HONcode criteria. However, it is necessary to educate the public about the evaluation tools of health websites to assess their credibility before using the provided content. In addition, improving readability will allow the public to read and understand the content.

## Appendix

Multimedia Appendix 1 Raw data of the cancer-related WeChat public accounts.

Multimedia Appendix 2 The HONcode principles.

Multimedia Appendix 3 The Suitability Assessment of Materials criteria and descriptions.

## Abbreviations

CWPA: cancer-related WeChat public account
HWPA: health-related WeChat public account
IARC: International Agency for Research on Cancer
SAM: Suitability Assessment of Materials
WCI: WeChat communication index
WPA: WeChat public account

## Data Availability

The data sets generated or analyzed during this study are available from the corresponding author upon reasonable request.
