# Supplementary material for: Evaluating the Quality of Cancer-Related WeChat Public Accounts: Cross-Sectional Study
Source: JMIR Cancer. 2024 May 30;10:e52156. doi: 10.2196/52156 (PMC11176876; doi:10.2196/52156)
Supplement: Multimedia Appendix 2 [file cancer_v10i1e52156_app2.docx]

1. Authoritative: indicate the qualifications of the authors

2. Complementarity: information should support, not replace, the doctor-patient relationship, the mission and the audience are explicated.

3. Privacy: Respect the privacy and confidentiality of personal data submitted to the site by the visitor

4. Attribution: Cite the source(s) of published information, date and medical and health pages

5. Justifiability: Site must back up claims relating to benefits and performance

6. Transparency: Accessible presentation, accurate email contact

7. Financial disclosure: Identify funding sources

8. Advertising policy: Clearly distinguish advertising from editorial content
