# Supplementary material for: Evaluating the Quality of Cancer-Related WeChat Public Accounts: Cross-Sectional Study
Source: JMIR Cancer. 2024 May 30;10:e52156. doi: 10.2196/52156 (PMC11176876; doi:10.2196/52156)
Supplement: Multimedia Appendix 3 [file cancer_v10i1e52156_app3.docx]

1.Content

a.Purpose is evident

b.Content about behavior

c. Scope is limited

d.Summary or review included

2. Literacy demand

a. Reading grade level

b.Writing style, active voice

c. Vocabulary uses common words d.Context is given first

e.Learning aids via"road signs"

3.Graphics

a.Cover graphic shows purpose b.Type of graphics

c.Relevance of illustrations

d.List,tables, etc. explained

e. Captions used for graphics

4. Layout and typography

a.Layout factors

b. Typography

c.Subheads(chunking) used

5.Learning stimulation, motivation

a.Interaction used

b.Behaviors are modeled and specific

c.Motivation-self-efficacy

6.Cultural appropriateness

a.Match in logic, language, experience

b.Cultural image and examples
